# The Moderating Role of Organizational Citizenship Behavior Toward Environment on Relationship Between Green Supply Chain Management Practices and Sustainable Performance

**DOI:** 10.3389/fpsyg.2022.876516

**Published:** 2022-05-30

**Authors:** Tamoor Azam, Saqib Yaqoob Malik, Diandian Ren, Wenrong Yuan, Yasir Hayat Mughal, Irfan Ullah, Muhammad Fiaz, Sidra Riaz

**Affiliations:** ^1^Department of Management Sciences and Engineering, School of Management and Economics, Kunming University of Sciences and Technology, Yunnan, China; ^2^Department of Business Management, University of Baltistan, Skardu, Pakistan; ^3^School of Banking and Finance, University of International Business and Economics, Beijing, China; ^4^Business School, University of International Business and Economics, Beijing, China; ^5^Department of Health Administration, College of Public Health and Health Informatics, Qassim University, Buraydah, Saudi Arabia; ^6^School of Management and Economics, Beijing Institute of Technology, Beijing, China; ^7^Department of Management Science, Qurtuba University of Science and Information Technology, Dera Ismail Khan, Pakistan; ^8^Department of Governance and Public Policy, National University of Modern Languages, Islamabad, Pakistan

**Keywords:** green supply chain management, organizational citizenship behavior for the environment, sustainability, natural resource-based view, partial least square structural equation modeling

## Abstract

The purpose of this study was to investigate the moderating role of “organizational citizenship behavior toward the environment” (OCBE) on the relation between “green supply chain management” (GSCM) practices and sustainable performance. The participants of the current study were manufacturing firms, and non-probability convenience sampling technique was used for selecting the sample size. The survey method used while data were collected from manufacturing firms was cross-sectional; a total of 151 responses were received and used in the analysis. For statistical analysis, “SmartPLS partial least square, structural equation modeling” PLS-SEM was used. From the findings, it was evident that in the measurement model, convergent and discriminant validities were established. In the second stage, a structural model was developed for hypotheses testing. It was found that sustainable performance was associated with proposed GSCM practices, excluding environmental management. OCBE being a moderator has significant impacts concerning GSCM operations and sustainability functions of firms. However, OCBE did not play the role of moderator between internal environmental management and EE and sustainable performance. The present study is significant for managers and policymakers for the attainment of competitive advantage, enhancement of OCBE, and sustainable performance. Furthermore, this research study is the first empirical study that has used OCBE as a moderator through the lens of NRBV.

## Introduction

Before the green initiatives and the sustainability concept were introduced, the World was considered a commodity and due to negligence and negative behavior of human beings and firms, environmental issues have increased in the past few decades (Mtutu and Thondhlana, 2016). Green practices in business operations, such as green supply chain management, can enhance sustainable performance (Saeed et al., 2018). In recent years, the concept of green practices has emerged and got the attention of organizations to control the issues related to the environment. These green initiatives have helped organizations to obtain a competitive advantage and sustainable performance (Cunningham, 2021). Green practices have improved the supply chain operations, businesses, and productivity of the firms. Organizations have introduced new terms such as green selection, green recruitment, green rewards and compensation, green policies and planning, green training and development, green performance evaluation, green workforce, green jobs and duties, green motivation (Siyambalapitiya et al., 2018), green branding of an employer (Tang et al., 2018), green marketing practices and green manufacturing (Luthra et al., 2016), green material sourcing (Eltayeb et al., 2011), green management (Khan et al., 2018), green supply chain management, green design, green distribution and warehousing (Khan and Qianli, 2017), green human resource management (Yuriev et al., 2018), and organizational citizenship behavior toward the environment (OCBE) to deal with environmental issues (Alt and Spitzeck, 2016). Indeed, researchers suggest that green practices in an organization are crucial for reducing environmental problems.

When employees help organizations to implement green policies and to obtain green objectives, the firms show their level of commitment and citizenship behavior toward the environment (Raineri and Paillé, 2016). The introduction of these new green terms, especially green supply chain management practices (GSCM), helps firms to attain sustainability. GSCM includes green purchase, green manufacturing, product packaging and distribution, internal environment management, environmental education, and investment recovery (Robertson and Barling, 2017). GSCM can help to reduce the cost of production, increase the satisfaction of customers, and improve the image and reputation of the firms by offering eco-friendly products and services (Luu, 2018). GSCM is getting attention not only in developed but in developing economies as well. OCBE is a new concept where employees take part voluntarily to contribute and offer their services for the betterment and improvement of their organizations without rewards (Mardani et al., 2020).

Khan and Qianli (2017) have figured out factors that help in achieving competitiveness. There are personal and organizational barriers to OCBE. Personal barriers include social norms, individual behavior, a lack of knowledge, awareness, and self-efficacy about environmental problems, while organizational barriers contain corporate values, absence of autonomy, lack of resources, and supervisor support (Çankaya and Sezen, 2019). Implementing OCBE in organizations helps managers and practitioners to increase sustainable performance (Alt and Spitzeck, 2016). The impact of OCBE on manufacturing firms and the relationship between the engagement of managers in OCBE and management practices of the environment are also substantial (Alt and Spitzeck, 2016). Training increases the awareness, importance, and significance of green objectives and OCBE (Pinzone et al., 2016). Past studies have reported the significant impact of green supply chain management practices and OCBE upon sustainable performance (Mavi and Standing, 2018). Having a green environment in the workplace is an essential factor for sustainable development (Luu, 2018). In this way, green activities give motivation to employees to pay more attention to eco-behavior, which influences OBCE and increase their concern about environmental protection to achieve sustainable performance. The present study has offered the following contributions toward the body of knowledge:

1. Literature regarding GSCM, OCBE, and sustainability practices is limited.
2. A research study on OCBE as moderator has not yet been reported.
3. Empirical evidence regarding GSCM and OCBE needs to be documented in Pakistan.
4. No study has presented such evidence from the context of Pakistan as provided in this article through the lens of the Natural Resource-Based View Theory (NRBV).

## Literature Review

### Sustainable Performance

For sustainability, the definition that is most commonly concurred is “development that meets the needs of the present without compromising the needs of future generations” (World Commission on Environment and Development (WCED), 1987). Previous research studies describe three dimensions of sustainability, that is, environmental performance, economic, and social performance (Chapman, 2021). Elkington (1994) has called sustainable performance (SP) as triple bottom line principle (Martens and Carvalho, 2017). Economic performance means dealing with financial matters, environmental performance helps to reduce issues related to the environment, and social performance shows how firms deal with employees, stakeholders, and wellbeing of employees, societies, and communities. Previous studies have highlighted the importance of sustainability in different business areas, such as green supply change management (Galbraith and Podhorska, 2021), project management (Inigo and Albareda, 2019), innovation (Neutzling et al., 2018), integrated management systems (Magon et al., 2018), and manufacturing (Yong et al., 2020). These studies have confirmed that for better performance, the initiative of green activities is essential (Choi and Hwang, 2015). Past studies have given more attention to economic and environmental performances but little focus is given to social performance, which is why this study has included all three dimensions of sustainable performance (Mathivathanan et al., 2018).

### Theoretical Basis for GSCM Practices

**Figure 1:**
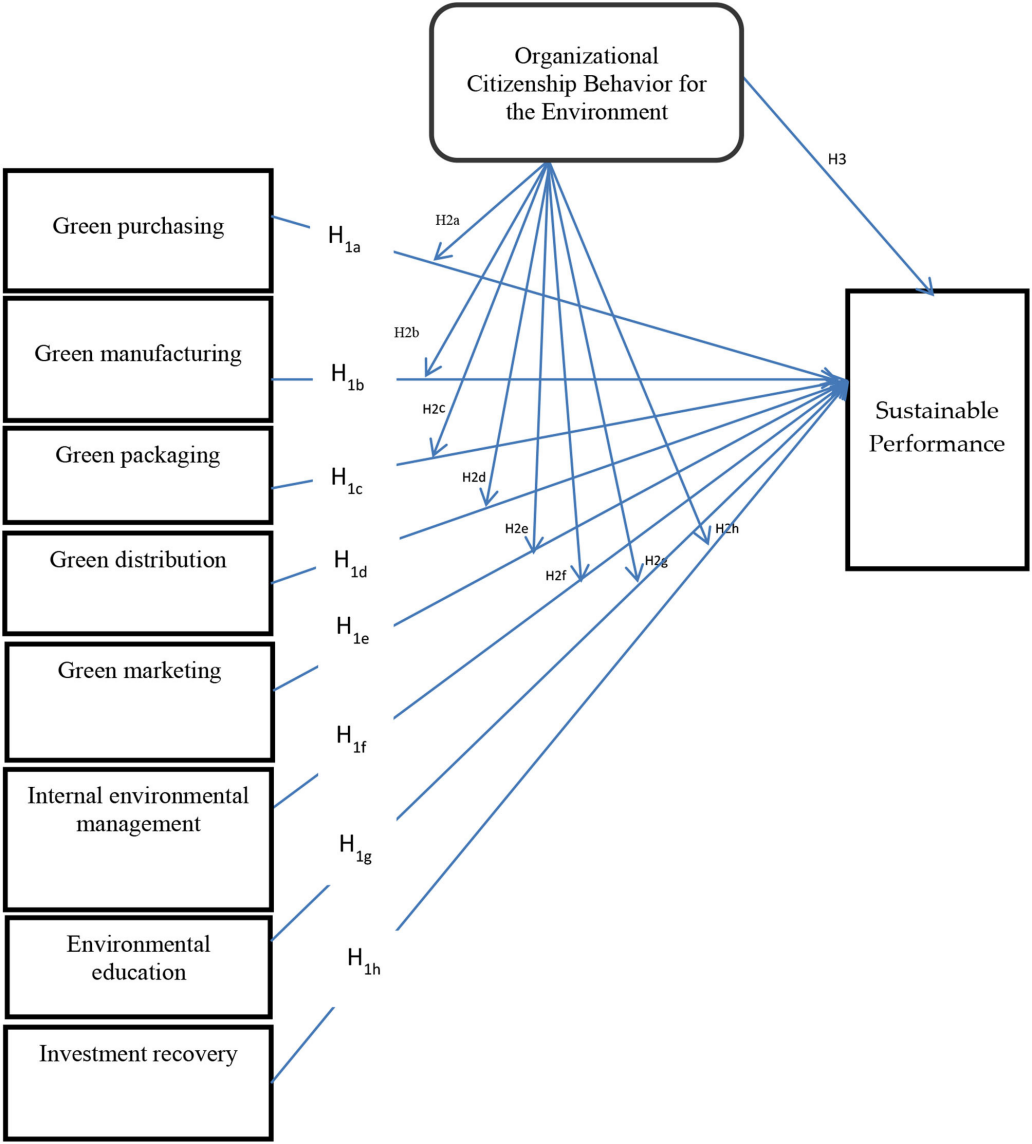
Conceptual model and hypotheses.

RBV theory was first introduced in 1991 but criticism has been raised that the environment is neglected in this theory. For this reason, Hart (1995) introduced a new theory called the natural resource–based view theory (NRBV), which covers the environmental issues caused due to human negligence. In addition, stakeholder theory (ST), resource dependence theory (RDT), resource orchestration theory (ROT), resource-based theory (RBT), sustainability and supply chain theory (SSCT), institutional and stakeholder theory (IST), contingency and production competency theory, green and coordination theory, strategic choice and resources based theory, knowledge-based view and goal setting theory, means–end theory, and stakeholder resource–based view theory (SRBV) have also been reported to contribute toward reducing environmental issues and increasing sustainable performance and organizational citizenship behavior toward the environment (Mardani et al., 2020) (see Figure 1).

### Green Supply Chain Management Practices

Green supply chain management includes green product design, purchase, distribution, processing, and handling all types of waste (Chu et al., 2017). GSCM can also be categorized into a series of practices focused on collaboration and evaluation to meet environmental and economic objectives (Chu et al., 2017). Many studies in the literature show that adopting GSCM practices can impact the environment positively and economic performance and cost-based performance (Ali et al., 2019). GSCM practices would allow businesses to have much more positive images in the minds of customers, the community, workers, and the government by reducing harm to the environment (Kovacova and Lǎzǎroiu, 2021). GSCM could lead to increased brand awareness, stronger stakeholder relationships, and strengthened motivation of employees (Geng et al., 2017). Firms have realized the importance of green initiatives and started implementing green policies to resolve environmental concerns in their supply chains (Cousins et al., 2019). At the same time, the study by Mardani et al. (2020) considered *eight* GSCM practices, including green purchasing, green marketing, green packaging, green manufacturing, green distribution, management of internal environmental, environmental awareness, and investment recovery (Suler et al., 2021). In particular, the current study emphasizes *eight* GSCM practices, as considered by Mardani et al. (2020) in their research study as mentioned above.

#### Green Purchasing

In the supply chain, the purchasing function is the first step. Green purchasing (GP) emphasizes coordinating with suppliers to manufacture goods that are environmentally friendly (Zhu et al., 2008). And green buying denotes the purchasing of goods and services that have a minimal or lower impact on human health and the environment (Foo et al., 2018). Through green purchasing, municipalities can minimize energy-related carbon emissions, solid waste, and a variety of other practices while increasing operational efficiencies. Selecting the perfect supplier has a huge impact on achieving the climate objectives of an organization (May et al., 2021). Green purchasing policies may increase the production of green goods and services and, through increasing demand, promote the growth of markets for products and services with environmentally sustainable characteristics. Findings indicated that green purchasing positively affects economic performance and environmental performance (Leal et al., 2020). The following hypothesis has been developed on the basis of the discussion above:

**H**_1a_**:** *Green purchasing has a positive effect on sustainable performance*.

#### Green Manufacturing

One of the significant milestones toward GSCM is green manufacturing (GM). It covers all sustainable activities that constantly integrate eco-friendly manufacturing methods for products. Green development was described by Vanalle et al. (2017) as a process of transformation that enhances resource utilization with high reliability and fewer ecological hazards. This is done by innovative product and process design. Chuang and Yang (2013) defined green manufacturing as manufacturing approaches that accept all aspects associated with environmental issues, constantly incorporating eco-friendly manufacturing processes (Eshikumo and Odock, 2017). The goal of green manufacturing is to minimize, monitor, prevent, and stop wastage during processing. Eshikumo and Odock (2017) identified green manufacturing as a transformational process that decreases the consumption of resources with high sustainability performance and limited ecological issues. The findings in the work of Tuwanku et al. (2018) showed a constructive association between green manufacturing and sustainable performance. For that reason, the following hypothesis is postulated:

**H**_**1b**_**:** *Green manufacturing has a positive effect on sustainable performance*.

#### Green Packaging and Distribution

Green packaging (GP) is not just an essential factor, but it is strongly tied to other aspects of the supply chain, and thus also as it has a significant influence on the environment (Kumar et al., 2017). Green packing refers to the marketing and usage of packaging, which results in better quality management (Kumar et al., 2017). Green packing involves cut downs in the volume, size, and weight of packaging and the usage of eco-sustainable materials. Thus, the subject of green packaging is also used to prove the company's contribution to the protection of the environment and to enhance brand recognition and reduce waste for packing of products by using colorful attractive wrappers, and so on (Maziriri, 2020). Green packaging is considered to convey an obligation to maintainability, environmental conservation practices, and green commodity characteristics in the market (Maziriri, 2020). The results of Maziriri (2020) showed green packaging's constructive influence on sustainable performance. And findings of Mardani et al. (2020) indicated that green packaging and distribution positively affect sustainable performance. The hypothesis is developed. The green distribution (GD) counts as a vital step that influences sustainability. Green delivery covers all operations during shipping to decrease environmental degradation and waste (Mwaura et al., 2016). According to Mwaura et al. (2016), transportation of products and services is essential in distribution. In designing green transportation, considerations such as diesel, transport modes, facilities, and operating practices are critical factors to remember (Lǎzǎroiu and Harrison, 2021). The fuel used by the transportation carrying the product, the pace of transportation, the distance to the consumer, and the features of the packaging (weight, shape, and material) affect the efficiency of green delivery (Mukonza and Swarts, 2020). Gasoline and diesel vehicles emit carbon dioxide, which causes global warming and acid rain. Therefore, companies can boost excellence by reducing waste and maximizing the available resources (Mukonza and Swarts, 2020). Hence, green packaging includes downsized packaging and the usage of green packaging materials (Mukonza and Swarts, 2020). The research study of Eneizan and Obaid (2016) came to the conclusion that the practice of green distribution positively affects the distribution activities of firms. And green distribution and packaging are positively related to sustainable performance (Mardani et al., 2020). Based on the above reasoning, the following hypothesis is established:

**H**_1c_**:** *Green packaging has a positive effect on sustainable performance*.

**H**_1d_**:** *Green distribution has a positive effect on sustainable performance*.

#### Green Marketing

Green marketing (GMRK) includes addressing human requirements with minimum adverse effects on the atmosphere and environment (Edsand and Broich, 2020). And green marketing is known as an organization's contribution to the development of safe, sustainable goods and services by the use of recyclable and quickly decomposable material use in packaging, upgraded methods of waste control, and more efficient use of resources (Liao et al., 2018). To improve their sales efficiency and eventually corporate image, firms adopt green marketing (Liao et al., 2018; Rath, 2013). The findings of Mardani et al. (2020) showed that green marketing positively impacts environmental performance. Furthermore, the study of Mardani et al. (2020) concluded that green innovation and green promotion positively influence a firm's performance. And research findings of Maziriri (2020) revealed that green advertising and green packaging have positively impacted the performance of SMEs (Ionescu, 2021). Thus, the following hypothesis was developed:

**H**_1e_**:** *Green marketing has a positive effect on sustainable performance*.

#### Internal Environmental Management

Internal environmental management (IEM) is the development of a company's internal environmental conservation policies and priorities related to the environment to ensure environmental safeguard (Kuo and Smith, 2018). IEM can be seen as a starting point as it relates to the devotion of top and middle management to being green, delivering staff training and designing initiatives for pollution control, having certifications, and implementing a continuous method of assessment (Kennedy and Hauslik, 2018). IEM encourages companies to pursue the targets set by top management and helps them to feel themselves part of GSCM as it tries to establish an atmosphere for learning, training, and being a specialist in management strategies relevant to sustainability (Boiral et al., 2018). Thus successful businesses concentrate on the IEM as a framework for the overall GSCM transition process. The study results of Boiral et al. (2018) confirmed that IEM positively influenced environmental performance. Furthermore, the findings of Mardani et al. (2020) showed that IEM positively affects sustainable performance.

H_**1f**_**:** *Internal environmental management has a positive effect on sustainable performance*.

#### Environmental Education

Environmental education (EE) applies to the environment in a systematic approach that is more human-oriented as interacting biophysical, social, economic, and political dimensions (Park, 2018). There is not a widely agreed definition of environmental education. However, the priorities and categories were set out by UNESCO in the late 1970s. Environmental education assists two main uses. First, to educate the employees about the environmental policy of the business, second to modify the individual behaviors of the employees to create a highly enduring and sustainable correlation with the environment (Park, 2018). Past findings of Mardani et al. (2020) disclosed that environmental education positively affects sustainable economic performance, and results of the study of Mesmer-Magnus et al. (2018) found that environmental education can encourage a high level of environmental awareness.

**H**_1g_**:** *Environmental education has a positive effect on sustainable performance*.

#### Investment Recovery

Investment recovery (IR) is known as yet another aspect and considered among the commonly examined factors in GSCM fields. IR is a green activity that includes restoring advantages from present investments that were formerly marked as waste (Anwar et al., 2020). Investment recovery is the ability of the firm to gain certain economic benefits (increase in revenue or reduced costs) from its environmental activities. Investment recovery deals with sales of surplus inventories, scrap and materials employed, and surplus capital resources as a strategic move for the achievement of the greatest value from its resources (Anwar et al., 2020). Therefore, these items may be safely retrieved or discarded. The past findings of Mardani et al. (2020) showed that investment recovery positively affects sustainable environmental and social performance.

**H**_**1h**_**:** *Investment recovery has a positive effect on sustainable performance*.

### Organizational Citizenship Behavior for the Environment

Organizational citizenship behavior for the environment (OCBE) is concerned with voluntary and rewarded-free environmental activities beyond the job requirements in an organizational context. Examples include searching for ways that make facilities and goods more sustainable or delivering environmental guidance to co-workers (Anwar et al., 2020). OCBE reflects the desire of workers to collaborate with their organization to carry out events that enable the natural environment beyond their obligations. Thus, OCBE refers to discretionary and environmentally friendly behavior. It will specifically help the company in minimizing environmental costs and increasing the environmental image of the organization (Anwar et al., 2020). The previous report focused largely on meeting renewable and low-carbon targets through the promotion and introduction of government policies (Malik et al., 2020) and green technology innovation (Malik et al., 2021). The role of active environmental behaviors of workers in the corporate green and low-carbon transformation has been overlooked by most research studies. The low-carbon production of companies does not depend entirely on the restrictions of hard rules but rather includes the active response and cooperation of employees. Hence, it is important to pay attention to employees' OCB. OCBE relates to discretionary and eco-friendly behaviors. This constructive employee conduct not only adds to organizational environmental efficiency but also bridges the environmental gap beyond the corporations' formal systems (Malik et al., 2021).

### The Moderating Role of OCBE

Organizational citizenship behavior for the environment covers several sustainable activities, containing regulation of workplace waste, recycling, and anti-carbon activities, and encouraging staff to pursue environment-friendly practices (Anwar et al., 2020). OCBE requires employees' voluntary acts and behaviors that are often contradictory to formal processes and benefits (Micheli et al., 2020). Not only does OCBE add greatly to the environmental efficiency of businesses, but it also improves their financial performance. OCBE has a positive impact on firms' environmental efficiency and will help them solve environmental issues, including global warming and climate change (Anwar et al., 2020; Malik et al., 2020). To our knowledge, this study was the first attempt to add OCBE as a moderator. No studies so far have taken OCBE as moderator. Consequently, the present research recorded empirical results of OCBE between green supply chain practices and sustainable performance. Therefore, it posits the following hypotheses:

H_2a_: OCBE moderates the relationship between green purchasing and SP.

H_2b_: OCBE moderates the relationship between green manufacturing and SP.

H_2c_: OCBE moderates the relationship between green marketing and SP.

H_2d_: OCBE moderates the relationship between green distribution and SP.

H_2e_: OCBE moderates the relationship between green packaging and SP.

H_2f_: OCBE moderates the relationship between internal environmental management and SP.

H_2g_: OCBE moderates the relationship between environmental education and SP.

H_2h_: OCBE moderates the relationship between investment recovery and SP.

### OCBE and Sustainable Performance

Organizational citizenship behavior for the environment is the voluntary and positive behaviors of workers at the workplace to contribute to the protection of the environment and benefit indirectly from the organization's environmental success and sustainability (Tuan, 2018). According to Tuan (2018), OCBE is an important component in the effective adoption of management of environmental programs and the alignment of environmental policy with workplace activities. Environmental protection has been one of the urgent priorities in society. For future generations, the protection of the natural eco-system and its resources is, therefore, becoming the main priority for managers and decision -makers (Chang et al., 2019). Hence, stakeholders urged stronger environmental awareness and responsibility from firms (Schmidt et al., 2017). As a result, conventional models are transformed into green models by incorporating sustainability strategies in their activities by firms (Malik et al., 2020). Such transformation of models can enhance competitive advantage (Malik et al., 2021) stated that OCBE includes the sense of sustainability inside and outside the firm that will help the firm attain its green objectives. Past studies have explored that OCB affects organizational performance (Roca-Puig, 2019). Several research studies have revealed an essential association between OCBE and environmental performance(Schmidt et al., 2017). OCBE also improves businesses' financial performance (Yusliza et al., 2020) and sustainable performance (Anwar et al., 2020). Therefore, the following hypothesis was established:

**H3:** *OCBE has a positive effect on sustainable performance*.

## Research Methods

### Data Collection Approach

The quantitative survey approach and instruments/questionnaires were adopted from previous studies. One-time data, that is, cross-sectional primary data, was collected and analyzed in PLS-SEM. Units of analysis were manufacturing firms. The population of the study was 700 manufacturing firms, including leather, plastic, cement, textile, beverages, agricultural products, sugar mills, and the construction industry. These firms are major contributors to the economy, and they are more exposed to environmental issues. The respondents/firms chosen for this study have already established their supply chain departments and know the environmental issues. Non-probability convenience sampling technique was used to select the sample size. Information about the firms was taken from firms listed on the Pakistan stock exchange. Respondents included supply chain managers, general managers, and directors.

### Measures/Instruments

The sustainability scale was adapted from Malik et al. (2020) and it comprised 15 items and five items for each construct, namely, social, economic, and environmental performance. This instrument was measured on 7-point scale (1 = not at all to 7 to a great extent). OCBE instrument was adapted from Anwar et al. (2020). It is a 10 items instrument, 3 for eco helping, 4 for eco civic engagement, and 3 for the eco initiative. The green supply chain management practices instrument was adopted from Çankaya and Sezen (2019), and it has 26 items scale. Three items for green purchasing and 3 items for green manufacturing, 4 items for internal environment management and item 4 is excluded for low factor loading, four items for green distribution and packaging, and 4 items for environmental education while items no 3 & 4 excluded for low loadings, six items for green marketing, and two items for investment recovery for economic performance 5 items were adopted but item no 2 & 3 excluded, for environmental performance 5 items were adopted and item 5 was excluded and all 5 items of social performance are retained. and all items of OCBE i.e. Eco helping, Eco civic engagement and Eco initiative are retained.

### Data Analysis Tools and Techniques

Partial Least Square Structural Equation Modeling PLS-SEM was used for the analysis of data. PLS-SEM is the best choice for researchers to analyze the data collected through surveys, that is, primary data. The first measurement model was developed and in that, the factor loadings must be >0.7, convergent validity (AVE and CR), AVE must be >0.5 and CR higher than >0.70. Discriminant validity was checked by the Fornellarcker criterion Square root of the AVE. Cronbach alpha must be >0.70. In the second stage, a structural model was developed to test the hypotheses in which bootstrapping 5,000 resample was run, beta values, *t*-statistics, *p*, BCIUL, and BCILL were used (Hair et al., 2014).

## Results

A total number of 700 firms were nominated as the participants of the study, from which 150 filled-in questionnaires were received and analyzed in the current study. The response rate was 21.4%. We covered all manufacturing sectors as mentioned in a previous section of this article, and respondents were selected on set criteria who had knowledge of GSCMP, OCBE, and already established their supply chain departments.

In PLS-SEM confirmatory factor analysis (CFA) was run to develop the measurement model. It was evident from findings (Table 1) that on the item of internal environment management, that is, item 4 was deleted from analysis owing to low factor loadings; two items from environmental education, that is, items 3 and 4, two items from economic performance, that is, items 2 and 3, one item from environmental performance, that is, item 5 were disqualified from analysis due to low factor loadings. It is also revealed that all items have factor loadings higher than 0.7 as suggested by Hair et al. (2014), but factor loadings of 0.6 for items of EE2, GM6, and eco initiative 2 were also retained in some cases as these values are approximately 0.7. Moreover, results revealed that all AVEs and CRs of all constructs met their threshold values, that is, >0.50 and >0.70 (see Table 1). Discriminant validity from Table 2—Fornel–Larcker criterion also revealed that constructs differ from each other. On the basis of the above discussion, it was assumed that convergent and discriminant validities are established and the scales used in this study are found reliable and valid.

**Table 1:** Measurement model.

**Variable**	**Items**	**Loadings**	**AVE**	**CR**	**Alpha**
Green purchasing	GP1	0.942			
	GP2	0.857	0.831	0.937	0.936
	GP3	0.934			
Green manufacturing	GM1	0.881			
	GM2	0.905	0.783	0.916	0.915
	GM3	0.869			
Green distribution and packaging	GDP1	0.934			
	GDP2	0.929	0.878	0.966	0.967
	GDP3	0.925			
	GDP4	0.960			
Internal environmental management	IEM1	0.943			
	IEM2	0.950	0.887	0.959	0.959
	IEM3	0.933			
	IEM4	-			
Green marketing	GM1	0.897			
	GM2	0.945			
	GM3	0.927			
	GM4	0.707	0.690	0.929	0.927
	GM5	0.783			
	GM6	0.684			
Environmental education	EE1	0.899			
	EE2	0.652	0.617	0.758	0.739
	EE3	-			
	EE4	-			
Investment recovery	IR1	0.962	0.906	0.951	0.951
	IR2	0.942			
Economic performance	EC1	0.810			
	EC2	-			
	EC3	-	0.708	0.879	
	EC4	0.881			
	EC5	0.832			
Environmental performance	EP1	0.896			
	EP2	0.884	0.702	0.904	
	EP3	0.843			
	EP4	0.717			0.968
	EP5	-			
Social performance	SC1	0.882			
	SC2	0.884			
	SC3	0.908	0.732	0.931	
	SC4	0.844			
	SC5	0.750			
Eco helping	Eco helping 1	0.809			
	Eco helping 2	0.884	0.686	0.867	
	Eco helping 3	0.789			
Eco civic engagement	ECE1	0.865			0.960
	ECE2	0.862	0.797	0.940	
	ECE3	0.915			
	ECE4	0.928			
Eco initiative	Eco initiative 1	0.936			
	Eco Initiative 2	0.699	0.621	0.828	
	Eco Initiative 3	0.705			

*Convergent Validity > AVE, Average Variance Extracted; CR, Construct/Composite Reliability Note Reliability for Sustainable performance and OCBE are taken as a whole*.

**Table 2:** Discriminant validity.

**Constructs**	**1**	**2**	**3**	**4**	**5**	**6**	**7**	**8**	**9**
Green distribution and packaging	**0.937**								
Green marketing	0.928	**0.830**							
Green purchasing	0.945	0.876	**0.912**						
Green manufacturing	0.994	0.930	0.999	**0.885**					
Internal environment management	0.977	0.934	0.932	0.986	**0.942**				
Investment recovery	0.942	0.915	0.908	0.960	0.953	**0.952**			
OCBE	0.937	0.970	0.906	0.947	0.945	0.962	**0.843**		
Environmental education	0.429	0.692	0.465	0.458	0.460	0.523	0.586	**0.786**	
Sustainable performance	0.956	0.941	0.946	0.975	0.955	0.951	0.969	0.553	**0.846**

*Bold values shows that these values are different form each other and met threshold values*.

Bootstrapping with resample rate of 5,000 was run in PLS-SEM to test the hypotheses. The structural model is presented in Table 3. To gain beta, standard error, *t*-statistics, significance values, BCIUL and BCILL bootstrapping was suggested by Hair et al. (2014). Three hypotheses, H2f, H1g, and H2g, were not supported while the remaining hypotheses got supported in this current study. The analysis of results revealed that green purchasing has an influential impact on sustainable performance (β = 0.205, *t* = 4.15, *p* < 0.05, and BCIUL = 0.306, BCILL = 0.110; see Table 3), and the findings revealed that one unit change in green purchasing could bring a 20.5% change in sustainable performance. Furthermore, it was also identified that green purchasing and OCBE (interaction term) also has a considerable impact on sustainable performance (β = 0.065, *t* = 2.528, *p* < 0.05, and BCIUL and BCILL both were positive), this moderation result explained that GP and OCBE together could bring 6.5% change in sustainable performance.

**Table 3:** Hypotheses testing moderation results.

**Hypothesis**	**Relationship**	**β**	**SE**	***t***	***p***	**BCIUL**	**BCILL**	**Support**
H1a	GP → Sustainable performance	0.205	0.050	4.15	0.000	0.306	0.110	Yes
H2a	GP*OCBE (Interaction term)	0.065	0.026	2.528	0.011	0.116	0.012	Yes
H1b	GM → sustainable performance	0.141	0.053	2.695	0.007	0.245	0.038	Yes
H2b	GM*OCBE (Interaction term)	0.182	0.048	3.720	0.000	0.280	0.091	Yes
**H1c–H1d**	GDandP → Sustainable performance	0.244	0.070	3.569	0.000	0.382	0.108	Yes
**H2c–H2d**	GDandP*OCBE (Interaction term)	0.130	0.029	4.582	0.000	0.187	0.075	Yes
H1f	IEM → Sustainable performance	0.098	0.061	1.692	0.045	0.198	0.002	Yes
H2f	IEM*OCBE (Interaction term)	0.027	0.019	1.517	0.065	0.055	−0.005	No
H1e	GMRK → Sustainable performance	0.089	0.052	1.737	0.041	0.174	0.004	Yes
H2e	GMRK*OCBE (Interaction term)	0.106	0.024	4.473	0.000	0.146	0.067	Yes
H1h	IR → Sustainable performance	0.112	0.040	2.896	0.004	0.192	0.035	Yes
H2h	IR*OCBE(Interaction term)	0.092	0.032	2.865	0.004	0.155	0.030	Yes
H1g	EE → Sustainable performance	0.038	0.024	1.517	0.129	0.087	−0.008	No
H2g	EE*OCBE(Interaction term)	0.000	0.018	0.149	0.882	0.033	−0.042	No
H3	OCBE → Sustainable performance	0.314	0.067	4.724	0.000	0.447	0.185	Yes

*Distribution and packaging are combined*.

*GP, Green purchasing; GM, Green manufacturing; GD and P, Green distribution and packaging; IEM, Internal environment management; GMRK, green marketing; IR, investment recovery; EE, environmental education; OCBE, Organizational citizenship behavior toward environment*.

Further analysis of findings revealed that green manufacturing has a positive and significant impact on sustainable performance (β = 0.141, *t* = 2.695, *p* < 0.05, and BCIUL and BCILL both were positive); this explains that one per cent change in green manufacturing could bring 14.1% change in sustainable performance. Likewise, the interaction term of GM and OCBE also revealed a positive and significant impact on sustainable performance (β = 0.182, *t* = 3.72, *p* < 0.05, and BCIUL and BCILL both were positive; see Table 3), 18.2% change in sustainable performance of the firms is possible due to green manufacturing and OCBE together. Moreover, green distribution and packaging also have a positive contribution to sustainable performance (β = 0.244, *t* = 3.569, *p* < 0.05, and BCIUL and BCILL were positive), 24.4% change occurs in sustainability due to green distribution and packaging (see Table 3). Similarly, GD&P, along with OCBE (interaction term), also has a significant role in sustainable performance (β = 0.130, *t* = 4.582, *p* < 0.05, and BCIUL and BCILL both were positive). This explains that due to OCBE and GD&P, 13% change occurs in the sustainable performance of the firms.

Similarly, hypotheses H1f and H1f were developed to investigate the internal environmental management (IEM) effect on sustainable performance. It was found positive and significant (β = 0.098, *t* = 1.69, *p* < 0.05, and BCIUL and BCILL both were positive), and the findings claimed that 9.8% of sustainable performance could be improved by internal environmental management. On the contrary, moderating effect of OCBE between IEM and sustainable performance is not significant (β = 0.027, *t* = 1.517, *p* > 0.05, and BCIUL was positive, but BCILL was negative). So this means there is no moderating role of OCBE between IEM and sustainability. Furthermore, the sustainable performance of the manufacturing firms was found to be positively and significantly predicted by green marketing (β = 0.089, *t* = 1.737, *p* < 0.05, and BCIUL and BCILL were positive). One unit change in green marketing could bring an 8.9% improvement in the sustainable performance of the firms. In the same way, OCBE has a significant moderating impact on green marketing and sustainable performance (β = 0.106, *t* = 4.47, *p* < 0.05, and BCIUL and BCILL were positive; see Table 3). It means a 10.6% increase in sustainable performance is due to green marketing and OCBE. In addition, investment recovery has significantly predicted sustainable performance (β = 0.112, *t* = 2.896, *p* < 0.05), and a 11.2% change in sustainable performance of the firms is due to investment recovery. Investment recovery includes resale of scrap material, used material, and excessive material. Investment recovery aims to recover the cost of surplus items or those items and products which are outdated and near to expiration. In addition, OCBE also moderated the relationship between investment recovery and sustainable performance (β = 0.092, *t* =2 .865, *p* < 0.05). Similarly, environmental education is not significantly related to sustainable performance as well as there is no moderating effect of OCBE on the relationship between environmental education and sustainable performance (β = 0.038, *t* = 1.57, *p* > 0.05, BCIUL = 0.87, BCILL = −0.008), (β = 0.000, *t* = 0.149, *p* > 0.05, BCIUL = 0.033, BCILL = −0.042). The last hypothesis was generated to examine the impact of organizational citizenship behavior on sustainable performance. There is a positive and significant impact of OCBE on sustainable performance (β = 0.314, *t* = 4.714, *p* < 0.05, BCIUL = 0.447, BCILL = 0.185). OCB is the voluntary behavior of employees and individuals, and this behavior is not affiliated with any formal rewards system of organizations. They participate in activities that help to reduce environmental issues.

## Discussion

This study has contributed toward the impact of green supply chain management practices on sustainable performance (economic, environmental, and social) with a moderating role of OCBE. The innovation of this study is based on the investigation of the correlation between green supply chain management practices and sustainable performance. To the best of researcher's knowledge, there is limited literature and empirical data available on green supply chain activities, sustainable performance, and environmental problems in manufacturing firms in Pakistan. In the context of natural resource-based theory, this study generated and examined the hypotheses that GSCM practices (green purchasing, green manufacturing, green packaging, green distribution, green marketing, internal environmental management, and investment recovery) have a positive association with sustainable performance (economic, social, and environmental). The findings indicated that only the EE attribute of GSCM practices has no positive and significant relationship with sustainable performance. In comparison, all other GSCM practices are significantly and positively related to sustainability performance. While OCBE being the moderator has positive and significant impacts between GSCM practices and sustainability performance of firms except for internal environmental management (IEM) and environmental education (EE). It was found that OCBE does not moderate IEM and EE, and sustainable performance.

The findings of this study show that six of the seven dimensions of GSCM practices are positively and considerably related to SP. The results of Vanalle et al. (2017) found that GSCM practices are found to be responsible for an improved economic, social, and environmental performance. The findings of the present study reveal that GSCM practices are positively and significantly related to SP. The findings of this current study support H1a. This explains that green purchasing is responsible for bringing significant sustainable performance in manufacturing firms. Green purchasing is positively related to sustainable performance. These findings were in line with the previous findings of Eshikumo and Odock (2017) who reported that GSCM practices, including green purchasing, have a positive effect on environmental and economic performance.

The findings of the current study show that green manufacturing is positively and significantly related to SP. The findings of this study underpin H1b. Green manufacturing involves all ecological concerns–related activities that continuously integrate environmental manufacturing processes. Green manufacturing is positively related to sustainable performance. These findings were consistent with the earlier findings of Mardani et al. (2020) showed that GSCM practices positively affect sustainable performance (social, environmental, and economic performance). The results of Tuwanku et al. (2018) indicated a positive relationship between green manufacturing and operational performance. Green manufacturing is a vital step in SCM activities. Hence, green processing focuses on producing environmentally friendly goods with limited assets.

H_1c_ and H_1d_ were developed to investigate the effect of green packaging and green distribution on SP. The findings of this study support H1c and H1d. “Green packaging” and “green distribution” were combined hence, retitled as “green packaging and distribution”, and it is the aspect that impacts environmental performance to the greatest degree. Green packaging and green distribution were positively related to sustainable performance. These findings were consistent with the previous findings of Mardani et al. (2020) showed that green packaging and distribution positively affect sustainable performance (social, environmental, and economic performance). The results of Maziriri (2020) showed green packaging positively influences business performance. In this study, it was found that green packaging and distribution positively influenced sustainable performance. Hence, for the firms in the manufacturing sector, green packaging and distribution is a valuable strategy, and the management has to be dedicated to getting sustainable performance with the evolving, changing modern marketing environment.

The current study findings support H1e. Green marketing is affirmatively related to sustainable performance. These findings were consistent with the earlier findings of Mardani et al. (2020) showed that green marketing has a positive impact on environmental performance but an insignificant impact on economic and social performance. As green marketing is mainly related to the promotion of products, fulfilling human requirements without damaging the environment, which is why it does not have any concern with the economic and social performance of firms.

And research findings of Maziriri (2020) suggested that green advertising and green packaging have a positive effect on the performance of SMEs. Green marketing is a significant strategy for an organization to build relationships with stakeholders. Thus, firms need to include more practical and coherent messages in their promotional activities so that they can increase sustainable performance. Companies have embraced green marketing to improve their company efficiency and, eventually, their corporate image. The findings of this study support the H1f. These findings were consistent with the previous findings of Mardani et al. (2020) showed that internal environmental management positively affects sustainable performance (environmental and social performance). Furthermore, the study results of Çankaya and Sezen (2019) confirmed that IEM positively influenced environmental performance. And a study by Anwar et al. (2020) revealed that internal environmental management increases economic performance. Based on the above discussion and findings, this study supported hypothesis H_1f_ that internal environmental management has a positive relationship to sustainable performance.

The findings of the present study showed that environmental education is not positively and significantly related to SP. H_1g_ was generated to investigate the effect of environmental education SP. Environmental education leads to a greater understanding of the environment and the achievement of green policy embraced by the organization. The findings of this study did not support the H1g. These findings supported previous findings of Edsand and Broich (2020) who reported the weak relationship between environmental education and renewable energy technologies, besides research findings indicated weak confirmation that environmental education could promote a higher degree of environmental awareness. Environmental education is not a method whose findings can be observed within a limited period. There are long-running mechanisms of self-sacrificing.

The findings of the present study showed that recovery of investment is positively and significantly related to SP. The findings of this study underpin the H_1h_. Investment recovery has a significant impact on SP. These findings were consistent with the previous findings (Çankaya and Sezen, 2019; Mardani et al., 2020) which showed that investment recovery positively impacts sustainable performance (environmental and social performance). And the study of Maziriri (2020) showed a significant relationship between investment recovery and environmental performance but was not related to financial performance. In the analysis performed, no significant relationship was explored between the recovery of investment and economic performance. This outcome is coincides with other studies showing that investment recovery is less attractive to developing countries (Vanalle et al., 2017).

Furthermore, hypotheses H_2a_, H_2b_, H_2c_, H_2d_, H_2e_, H_2f_, H_2g_, and H_2h_ were generated to investigate whether OCBE is moderated between GSCM practices and sustainability performances. The results revealed that OCBE positively and significantly moderated the relationship between GSCM practices including (green purchasing, green manufacturing, green packaging, green distribution, green marketing, and investment recovery) and sustainable performance while OCBE is not moderated significantly among internal environmental management and environmental education and sustainable performance as it has been mentioned in the literature review section that to our knowledge, this study was the first attempt to add OCBE as a moderator. No studies have taken OCBE yet as moderator. So, this study offers an innovative contribution to the body of existing literature as a research study on OCBE as a moderator has not yet been reported. Thus, on the basis of our results, hypotheses H_2a_, H_2b_, H_2c_, H_2d_, H_2e_, and H_2h_ have been accepted, and H_2f_ and H_2g_ are rejected. The moderating variable OCBE has considerably and in a positive way moderated the relationship between GSCM practices sustainable performance. Additionally, it indicates that the effective implementation of GSCM practices can enhance sustainable performance. Present research recorded empirical results of OCBE between green supply chain practices and sustainable performance.

Furthermore, H_3_ was generated to observe the impact of OCBE on SP. Research has also found that followers are more pertinent to partake in OCBEs if firms have a positive and constructive attitude toward the environment (Tuan, 2018). The study findings support the H3. OCBE has a significant impact on SP. These findings supported earlier findings of Dabija et al. (2018) and Malik et al. (2021), which showed a positive effect of OCBE on environmental performance, and also the findings of Schmidt et al. (2017) indicated that OCBE is essential in the green product development performance of firms. Similarly, this study got support from the findings of Roca-Puig (2019) reported a positive and significant impact of GSCM practices on a firm's performance. OCBE serves as a motivational strategy in the firm to capture pro-environmental behaviors, and these behaviors are irreplaceable because of individuals' varied ability to contribute successfully to environmental action (Roca-Puig, 2019). The findings indicated that all the GSCM practices are significantly and positively related to sustainability performance. Only EE attributes of GSCM practices found an insignificant relationship with sustainability; hence it needs further examination in upcoming studies to offer innovative insights in the area of GSCM through NRBV. Besides, the natural resource-based view theory has extended the body of knowledge of GSCM (NRBV) in the manufacturing sector in Pakistan's scenario.

## Conclusions

It is concluded from the above discussion that manufacturing firms have to select suppliers who meet the criteria of their green initiatives. In addition, suppliers have to fulfill the standard criteria of green objectives set by manufacturing firms. Furthermore, it is concluded that setting green objectives helps firms to attract potential suppliers and loyal customers, and it increases the image of the firms in eyes of stakeholders, suppliers, societies, and communities. More investors will be attracted to the firms and they will be able to expand their business. Moreover, it is also concluded that initiating and implementing green activities help the firms to save cost, packaging and transportation cost, save the environment, reduce eco problems, produce eco-friendly products and services, and obtain a competitive advantage and sustainable performance over competitors. For the implementation of the green activities, budget and capital are needed, which will be a cost/financial burden for some manufacturing firms but in the long term, it will have benefits for firms and also for stakeholders and societies. Customers are showing more concern for eco-friendly products and for this purpose they are willing to pay more for eco-friendly products so firms should implement green activities and motivate their employees to help achieve their objectives through the use of OCBE.

### Theoretical Implications

This study has expanded the existing literature and body of knowledge of GSCM, OCBE, and sustainable performance. Through the findings of this study, it is concluded that natural resources–based view theory, stakeholder resource—based view theory, stakeholder theory, resource-based view theory, resource dependence theory (RDT), resource orchestration theory (ROT), resource-based theory (RBT), sustainability and supply chain theory (SSCT), institutional and stakeholder theory (IST), contingency and production competency theory, green and coordination theory, strategic choice and resources based theory, knowledge-based view and goal-setting theory, means–end theory, and stakeholder resource–based view theory (SRBV) are helpful to obtain competitive advantage (Mardani et al., 2020).

### Practical and Policy Implications

This article has offered implications for practitioners in firms as well as those who are involved in policy making. While making policy, no one can neglect the environment in the manufacturing sector. This study has offered a win–win principle for business, the environment, and societies as well. It has offered profit/economic environmental and social benefits. Managers must do cost–benefit analysis because at the initial stage by initiating green activities some costs such as operational, training procurement, and investment would be increased but on the other hand, there are some economic benefits such as green production/manufacturing, green packaging and distribution, and investment recovery offers some economic benefits to the firms.

### Limitations and Future Directions

Despite the many contributions of the current study, there are certain limitations to this study that are important to report. This study has been conducted in a manufacturing sector but the same model and theories could be applied to retailers and wholesaler type of organizations also. Although data were collected from relevant people in manufacturing firms and the reliability and validity of data are established by using advanced tests, namely, the measurement model in PLS-SEM but the data used in the study are cross-sectional, and causal inference is not possible to explain due to cross-section structure of the data/study, therefore, future studies can use longitudinal or mix method research. As suggested by Çankaya and Sezen (2019), this study used OCBE as a moderator. It is suggested that in future studies, the moderating role of green intellectual capital may be investigated between GSCM practices and sustainability. Moreover, OCBE, green HRM, and CSR can be used as mediators among GSCM practices and sustainability performance by using other relevant theories to extend the body of knowledge and answer future research questions such as whether supply chain responsibility is a missing link between green supply chain management practices and sustainable performance.

## Data Availability Statement

The original contributions presented in the study are included in the article/supplementary material, further inquiries can be directed to the corresponding author/s.

## Ethics Statement

The studies involving human participants were reviewed and approved by University of Sciences and Technology, Yunnan, China. The Ethics Committee waived the requirement of written informed consent for participation.

## Author Contributions

TA, SM, and YM did the conceptualization, writing manuscript, and analysis of data. While DR, WY, and IU helped in data collection and incorporating all suggestions. MF and SR helped in proof reading and editing of manuscript.

## Funding

This research article was supported by the Fundamental Research Fund for the Central Universities in UIBE (Grant No. 20YQ14).

## Conflict of Interest

The authors declare that the research was conducted in the absence of any commercial or financial relationships that could be construed as a potential conflict of interest.

## Publisher's Note

All claims expressed in this article are solely those of the authors and do not necessarily represent those of their affiliated organizations, or those of the publisher, the editors and the reviewers. Any product that may be evaluated in this article, or claim that may be made by its manufacturer, is not guaranteed or endorsed by the publisher.
